# Is the distribution of care quality provided under pay-for-performance equitable? Evidence from the Advancing Quality programme in England

**DOI:** 10.1186/s12939-016-0434-5

**Published:** 2016-09-23

**Authors:** Thomas Mason, Yiu-Shing Lau, Matthew Sutton

**Affiliations:** Centre for Health Economics, University of Manchester, Manchester, UK

**Keywords:** Health inequalities, Economics, Health policy, Health Services, NHS

## Abstract

**Background:**

The limited number of existing previous studies of the distribution of quality under NHS Pay-for-performance (P4P) by income deprivation have not analysed the relationship at the individual level and have been restricted to assessing P4P in the primary care setting. In this study, we set out to examine how achievement of P4P 'quality measures' for which NHS hospitals were paid was distributed by income deprivation.

**Methods:**

*Design*: Retrospective analysis of performance data reported by hospitals, examining how the probability of receiving 23 indicators varied by patients’ area deprivation using logistic regression controlling for age and gender. *Sample*: We use anonymised observational data on 73,002 patients admitted to hospitals in the North West of England between October 2008 and March 2010 for the following five reasons: acute myocardial infarction; coronary artery bypass grafting; heart failure; hip and knee replacement; and pneumonia.

**Results:**

The relationship between quality and deprivation varies depending on the point of delivery in the treatment pathway, and on whether delivered for conditions in scheduled or unscheduled care. For diagnostic tests on arrival, receipt of quality was: pro-rich in scheduled care and pro-poor in unscheduled care. Receipt of quality was pro-poor for pre-surgery measures in scheduled care. Receipt of quality at discharge was pro-rich.

**Conclusion:**

Unlike in primary care, in secondary care quality is not systemically distributed by income deprivation under P4P. Whilst improvements in health inequalities are important system objectives; they may not necessarily be achieved by the adoption of P4P schemes in hospitals.

## Background

Various ‘pay-for-performance’ (P4P) initiatives have been adopted in health care across the world. Typically, the primary objective of these initiatives is to improve the quality of care provided to patients and thus improve health outcomes. Prior to 2008, experiments with P4P in the National Health Service (NHS) in England have been limited to a single scheme which was adopted in the primary care setting: the Quality and Outcomes Framework (QOF).

Generally, studies evaluating the impact of P4P schemes have focused on the quality of care provided under P4P [1–6]. In addition, there are a limited number of analyses of the impact on health outcomes [7–9]. A recent Cochrane review found no evidence that financial incentives improve health outcomes [10]. Comparatively limited evidence exists in relation to the impact of P4P on the distribution of quality [11]. Many health care systems state the reduction of health inequalities as a policy objective [12] and so the potential distributional impacts of P4P schemes are an important consideration for policymakers.

Whilst the majority of the P4P literature focuses on the relationship between P4P and quality of care, previous studies also exist which have considered the distribution of quality under P4P. A recent systematic review of the link between P4P and equity [11] found 22 relevant articles, noting that whilst the international ‘distributional’ evidence base is limited, a growing evidence-base exists on the distribution of quality under P4P in the primary care setting in the English NHS [13–19]. These studies comprise observational analyses of the QOF scheme which was introduced in 2004 in the NHS and rewarded providers of primary care services for achievement on a large number of quality indicators. The majority of these studies focused on the relationship between receipt of quality indicators and area deprivation, with a consensus of studies finding a significant relationship between relatively higher deprivation and a lower probability of receiving quality – the gap narrowed over time [16] but there is no evidence that the QOF reduced health inequalities [20].

In this paper, we provide the first analysis of the distribution of quality under P4P in NHS secondary care. Equity under P4P may be different in the secondary care setting: patients are more likely to be in seriously-ill health and communication between the patient and the health care professional is relatively brief (compared to primary care). This analysis will allow us to provide details as to what is the current distribution of care quality provided under P4P in a hospital setting.

We use data from the first P4P scheme introduced in hospitals in England. We use, unlike many studies, data at the level of the individual and our dataset contains 349,862 observations on 73,002 individuals.

### The incentive scheme

The Advancing Quality (AQ) initiative was the P4P scheme adopted in secondary care for the NHS - it was introduced in October 2008 in all 24 NHS hospitals in the North West of England that provide emergency care. We provide details on the characteristics of these hospitals in Table 1. The participating hospitals were required to collect and submit data on 23 indicators relating to five health conditions in total – two in scheduled care: hip and knee replacement (HK) and coronary artery bypass grafting (CABG). We have data on three conditions in unscheduled care: acute myocardial infarction (AMI); pneumonia (PN) and heart failure (HF).

**Table 1:** Characteristics of participating hospitals

Characteristic	Number	Percentage
Scale and scope of hospital		
Teaching or specialist	5	21
Large general	7	29
Medium general	8	33
Small general	4	17
Foundation Trust Status ^a^		
Non-foundation Trust	17	71
Foundation Trust	7	29
Rating of overall quality of care in 2007^b^		
Excellent	7	29
Good	13	54
Fair or weak	4	17
Rating of financial management in 2007^c^		
Excellent	11	46
Good	7	29
Fair or weak	6	25

^a^Foundation trust hospitals that have been approved by the national regulator to have additional managerial and financial autonomy; classified according to status in 2007

^b^The rating represent the composite rating of performance in 2007 by the national regulator (the Healthcare Commission) against core standards, existing national targets, and new national targets for quality

^c^The rating represents the composite rating of performance in 2007 by the national regulators (the Healthcare Commission and Monitor) on financial standing, management, and control

Financial rewards were based on the relative performance of the participants. At the end of the first year, participants whose performance was in the top quartile for each health condition received a bonus payment equal to four percent of the revenue they receive for the patient activity for this condition. The next-best performing quartile received bonus payment equal to two percent of revenue. The remaining participants did not receive bonus payments. There were no penalties for relatively poor performance. The total value of bonuses paid to participating hospitals in the first year was £3.2 million.

When AQ was first introduced, Chief Executive Officers (CEOs) of hospitals collectively decided to assign bonuses to the clinical teams responsible for earning them. These payments could not be taken as personal income; instead they would be reinvested into improving clinical quality.

## Methods

The rationale for adopting P4P is that it changes the behaviour of providers. Providers might be motivated to exert greater effort for tasks that can be completed at the lowest effort and financial cost in order to maximise the gap between revenue and costs [21]. This may mean that, under P4P, providers select types of tasks and/or patients for with a higher expected revenue-cost differential: such as patients from less deprived backgrounds who typically have fewer co-morbidities and are therefore simpler/less costly to treat [22].

Conversely, it may be that providers do not target ‘simpler’ patients, but that P4P induces providers to treat more complicated patients who might be neglected in its absence [16].

We obtained a unique patient level dataset from the North West Advancing Quality team. This dataset is an extract from Secondary Uses Service (SUS) of AQ qualifying patients using ICD10 codes (International Classification of Diseases version 10) and procedure codes. These data cover the time period October 2008 to March 2010. The data are merged at an individual level with the Quality Measures Reporter (QMR) which contains the information entered by the provider on patients’ receipt of clinical process measures.

We also use the income deprivation score extracted from The English Indices of Deprivation 2010. The index of income deprivation (IMD 2010) is a lower level super output area (LSOA) measure of income deprivation. We have 4749 LSOAs in our sample, and on average there are 15 individuals in each LSOA. This measure of income deprivation reflects the proportion of the LSOA population in receipt of state benefits and tax credits on grounds of low income (IMD 2010). Patients are assigned a deprivation score based on their area of residence. The mean deprivation score in our sample is 0.182 and the standard deviation is 0.133 (i.e. on average in our sample, 18.2 % of each LSOA population is in receipt of state benefits and tax credits on grounds of low income; and the average deviation around the mean is 13.3 %).

Our data contain 349,862 observations on 73,002 patients for five conditions: 11,889 AMI patients; 2989 CABG patients; 10,791 HF patients; 21,436 HK patients and 25,897 PN patients.

We model the probability that an individual received a quality indicator based on their LSOA using logistic regression, standardising for age and gender.

## Results

We present the Marginal Effects (ME) of a change in LSOA on individuals’ probability of receipt of quality measures (Tables 2 and 3). The MEs show the change in a patient’s probability of receipt of an indicator for residing in an area with an additional 10 % of the population in receipt of social security payments on the basis of low income. When referring to ‘poorer’ patients (below), we mean patients residing in an area with an additional 10 % of the population in receipt of social security payments on the basis of low income.

**Table 2:** Effect of deprivation status on the probability of receiving quality indicators (Scheduled Care)

Pathway point	Condition	Scheduled care: indicator definition	Marginal Effect (ME)	Std. Error
Arrival (Test)	HK	Prophylactic antibiotic selection for surgical patients	−0.0879***	−0.0198
Arrival (Test)	CABG	Prophylactic antibiotic selection for surgical patients	−0.0554	−0.029
Pre-surgery	HK	Prophylactic antibiotic less than 1 h prior to surgical incision	0.0244	−0.0199
Pre-surgery	CABG	Prophylactic antibiotic less than 1 h prior to incision	0.0173	−0.0276
Pre-surgery	HK	Recommended venous thromboembolism prophylaxis ordered	0.0401**	−0.0137
Pre-surgery	HK	VTE Prophylaxis under 24 h pre- or post-surgery	0.1226***	−0.0171
Post-surgery	HK	Prophylactic antiobiotics discontinued under 24 h post-surgery	−0.0169	−0.0125
Post-surgery	CABG	Prophylactic antiobiotics discontinued under 24 h post-surgery	−0.01	−0.0339
Discharge	CABG	Aspirin prescribed at discharge	−0.0271	−0.0279

(The MEs show the change in a patient’s probability of receipt of an indicator for residing in an area with an additional 10 % of the population in receipt of social security payments on the basis of low income); *** *p* < 0.001; ** *p* < 0.01; *p < 0.05

**Table 3:** Effect of deprivation status on the probability of receiving quality indicators (Unscheduled Care)

Pathway point	Condition	Unscheduled care: indicator definition	Marginal effect (ME)	Std. Error
Arrival (Test)	HF	Evaluation of LVS Function	0.009	−0.0262
Arrival (Test)	PN	Initial antibiotic selection for CAP in immunocompetent patients	0.0593*	−0.029
Arrival (Test)	PN	Blood cultures in A&E before antibiotic administration	0.1027*	−0.0507
Arrival (Prevention)	AMI	Aspirin at arrival	0.0018	−0.0144
Arrival (Prevention)	AMI	Fibrinolytic Therapy within 30 min of arrival	0.0184	−0.0954
Arrival (Prevention)	PN	Initial antibiotic within 6 h of arrival	−0.049	−0.0332
Discharge	AMI	Adult smoking cessation advice	−0.1521**	−0.0561
Discharge	HF	Discharge instructions	−0.1530***	−0.0394
Discharge	HF	Adult smoking cessation advice	−0.0849	−0.1154
Discharge	PN	Adult smoking cessation advice	−0.1002	−0.0554
Discharge	AMI	Aspirin prescribed at discharge	0.0066	−0.0124
Discharge	AMI	ACEI or ARB for LVSD	0.026	−0.0326
Discharge	HF	ACEI or ARB for LVSD	−0.0086	−0.0395
Discharge	AMI	Beta blocker prescribed at discharge	−0.0026	−0.0237

(The MEs show the change in a patient’s probability of receipt of an indicator for residing in an area with an additional 10 % of the population in receipt of social security payments on the basis of low income); *** *p* < 0.001; ** *p* < 0.01; *p < 0.05

In scheduled care, we found statistically significant relationships for three out of nine indicators. Poorer patients were found to have a higher probability of receiving pre-surgical interventions for both hip and knee replacements and coronary artery bypass grafting (CABG) (Table 2). A statistically significant relationship with income deprivation exists for two of these pre-surgical measures: being ‘poorer’ increased patients’ probabilities of having venous thromboembolism prophylaxis (VTE) ordered by 0.4 % (*p* < 0.05); and increased patients’ probabilities of receiving prophylaxis within 24 h of surgery by 1.2 % (*p* < 0.01).

Diagnostic testing on arrival prior to scheduled procedures was ‘pro-rich’. For hip and knee replacement, the probability of being given the diagnostic test for prophylactic antibiotic selection was 0.8 % lower (*p* < 0.01) for poorer patients. The relationship was also negative (0.06 %; *p* > 0.1) for the same diagnostic testing for CABG patients.

For our three conditions in unscheduled care we found significant relationships with income deprivation for four out of 14 indicators. In the case of AMI, the probability of receiving smoking cessation advice on or prior to discharge from hospital was 1.52 % lower for poorer patients (*p* < 0.05). For a similar discharge indicator for heart failure patients (giving specific discharge instructions) we found a comparable effect both in the direction and magnitude of the effect: poorer patients’ probabilities of receiving discharge advice were 1.53 % lower (*p* < 0.01).

We found two further significant relationships for pneumonia patients – both for quality indicators which represent diagnostic tests carried out for patients on arrival at hospital. Poorer patients had a 0.59 % higher (*p* < 0.1) probability of receiving a diagnostic test for antibiotic selection and had a 1.03 % higher (*p* < 0.1) probability of having blood taken in accident and emergency prior to the receipt of antibiotics.

## Discussion

This study provides the first analysis of P4P on distribution of health care in hospitals situated in a public system with universal coverage. We utilize hitherto unexploited cross-sectional, patient-level data and control for covariates such as age and gender.

Whilst the QOF literature suggests that, under P4P, the distribution of quality was skewed towards richer patients in the primary care setting, the results in this study are equivocal when taken at face value. There is no evidence of a systematic relationship across all of the quality indicators.

However, we found that pre-surgical measures in scheduled care were more likely to be received by patients from areas of lower income deprivation. We also found a ‘pro-poor bias’ for two diagnostic tests on arrival in pneumonia patients. Conversely, we found that testing on arrival for hip and knee patients was significantly ‘pro-rich’ for one indicator (prophylactic antibiotic selection).

For many of our indicators, we did not find a statistically significant result. This may suggest that, in these cases, there was no relationship between income deprivation and the probability of the receipt of quality. It may also be the case that receipt of certain indicators is driven more extensively by exogenous factors beyond the control of providers/health professionals than for others.

The evidence base from the previous P4P initiative adopted in the NHS (the QOF) suggests that, under P4P, quality was distributed towards patients from richer areas, but that the ‘pro-rich’ bias narrowed over time. However, such a scheme had not been adopted in the secondary care setting in the NHS prior to the AQ initiative. Policymakers’ interest in P4P lies both in its capacity to improve the quality of care delivered; and its impact on wider system objectives such as the reduction of health inequalities. In light of this, it is also important to understand the differential distributional impact P4P can have in the alternative settings of primary care and hospitals.

This study is subject to some (but not all) of the same limitations as the general P4P evidence base. We have no pre-AQ data and this means we only observe the distribution under the P4P scheme – we cannot compare pre-intervention with post-intervention.

We found that pre-surgical measures in scheduled care were more likely to be received by patients from areas of lower income deprivation. This may be the consequence of the strategic targeting of preventative pre-surgical measures: poorer patients are typically higher risk, and these measures are conventionally targeted at higher risk groups. The results for ‘testing on arrival’ measures were divergent: pro-poor in unscheduled care (where processes are typically performed more hastily), and pro-rich in scheduled care.

## Conclusions

The results of our study diverge from the QOF literature as we find that the distribution of quality under P4P was not systematically skewed across income deprivation. Instead, it may be that our findings suggest that the distribution of quality under P4P depends crucially on the context – whether in primary or secondary care; varying depending on the point in the treatment pathway; and depending on the extent to which the receipt of the indicator is determined by exogenous factors beyond the control of providers. Nevertheless, it is difficult to draw definitive conclusions regarding P4P schemes effects on equity; further research is required. But policymakers may still wish to be cautious when considering adopting and designing P4P schemes as their effects are unpredictable, the evidence base is ambiguous and their effects may be in direct contradiction to health system objectives: health equity is an important system objective that may necessarily not be achieved by payment-for-performance. Policymakers may also wish to heed to following advice: when it comes to P4P schemes and their unpredictable distributional impacts, the devil is in the detail.

**Table:** 

**Policy Implications:**
• The distributional effects of P4P depend crucially on contextual factors such as: whether in scheduled or unscheduled care; the point of delivery in the treatment pathway; incentive structures • Incentive schemes are unlikely to yield a uniform distributional results across different indicators • Health equity is an important system objective that may not be achieved by payment-for-performance.

**Table:** 

**What this paper adds:**
• We provide the first analysis of the distribution of quality under P4P in hospitals in a public health care system with universal coverage • Findings are obtained from a large dataset (as compared to existing studies) • We use data at the patient level (349,862 observations on 73,002 individuals)

**Table:** 

**What is already known:**
• P4P has been adopted in various context in many health system and there is not a clear and established consensus as to its effects generally – but more specifically in relation to health inequalities • In NHS primary care, the QOF literature suggests that quality was distributed towards richer patients under P4P • Previous studies of NHS P4P and its deprivational distribution have not analysed the relationship at the individual level and have been restricted to assessing P4P in the primary care setting

## Abbreviations

NHS: National Health Service
P4P: Payment-for-performance
QOF: Quality and Outcomes Framework
AQ: Advancing Quality
HK: Hip and knee
CABG: Coronary artery bypass grafting
AMI: Acute myocardinal infarction
PN: Pnuemonia
HF: Heart failure
CEO: Chief Executive Officer
SUS: Secondary Uses Service
ICD10: International Statistical Classification of Diseases and Related Health Problems version 10
QMR: Quality measures reporter
IMD: Index of multiple deprivation
LSOA: Lower super output area
ME: Marginal effect
VTE: Venous thromboembolism prophylaxis
